# Efficacy of the microscopic parachute end-to-side technique for creating large-to-small venous anastomoses in free flaps in the extremities

**DOI:** 10.1016/j.jpra.2022.10.003

**Published:** 2022-10-08

**Authors:** Makoto Motomiya, Naoya Watanabe, Mitsutoshi Ota, Kohei Shimoda, Daisuke Kawamura, Norimasa Iwasaki

**Affiliations:** a Department of Orthopaedic Surgery, Obihiro Kosei Hospital Hand Center, Obihiro, Japan; b Department of Orthopaedic Surgery, Higashisaitama General Hospital, Satte, Japan; c Department of Orthopaedic Surgery, Faculty of Medicine and Graduate School of Medicine, Hokkaido University, Sapporo, Japan

**Keywords:** End-to-side anastomosis, Free flap, Microscopic parachute end-to-side (MPETS) anastomosis, Parachute, Venous anastomosis, Venous valve

## Abstract

**Purpose:**

The availability of reliable and suitably sized veins is limited for creating free flaps to treat severe trauma and infection, and it is important to manage vessel size discrepancy between the recipient and flap veins. We evaluated the clinical outcomes of free flaps with large-to-small venous end-to-side (ETS) anastomoses using the microscopic parachute end-to-side (MPETS) anastomosis in soft tissue defects in the extremities. This procedure comprises mainly a wide-slit venotomy and parachute procedure at the heel.

**Methods:**

We examined 24 free flaps in 23 patients given a large-to-small venous anastomosis using the MPETS technique. Patient demographics, details of vessel anastomoses, and flap outcomes and complications were obtained from medical records.

**Results:**

Two veins were anastomosed in six flaps. Thirty anastomosed veins were assessed, and 24 deep veins, all of which accompanied main arteries, were chosen as recipient veins. The mean diameters were 1.5 mm in the recipient veins and 2.7 mm in the flap veins, and the mean vessel size discrepancy was 1.8-fold (range 1.3–3.3 fold). Because of the presence of venous valves at the anastomotic site, trimming of venous cusps was performed in six veins. All flaps survived, though one venous thrombosis occurred because of pedicle kinking in a case with a short pedicle.

**Conclusions:**

The MPETS technique is simple, reliable, and useful for performing various types of venous anastomoses regardless of a vessel size discrepancy and the presence of a venous valve. This may be a good option for large-to-small venous anastomosis in free flaps.

## Introduction

Venous thrombosis is one of the most critical causes of free flap failure.^1^, ^2^, ^3^ Although large superficial veins are likely to be used as recipient veins, superficial veins may be damaged or included in the zone of injury and may be unreliable for soft tissue reconstruction to repair traumatic injury or after severe infection.^4^^,^^5^ Some surgeons have suggested that deep veins accompanying major arteries are likely to avoid injury and may be more reliable as recipient vessels than superficial veins.^6^^,^^7^ However, deep veins selected as the recipient veins often have a small diameter, and the surgeon sometimes struggles when performing a complicated anastomosis of large flap veins to small recipient veins (large-to-small anastomosis).^8^^,^^9^ This venous size discrepancy is thought to influence thrombus formation,^2^^,^^10^^,^^11^ and it can be difficult to select the optimal recipient veins and perform venous anastomosis in cases with a vessel size discrepancy in a free flap.

Although the end-to-side (ETS) technique is useful in anastomoses with a vessel size discrepancy in free flaps,^12^, ^13^, ^14^ this technique is considered to be more difficult than the end-to-end (ETE) technique. The ETS technique is difficult especially in cases requiring a large-to-small venous anastomosis.^9^ We use the microscopic parachute end-to-side (MPETS) anastomosis, which is a modification of the ETS technique commonly used by cardiovascular surgeons for venous anastomoses in free flaps.^15^^,^^16^ The MPETS technique provides a good view inside the lumen of the recipient veins through the wide-slit venotomy and allows an easy anastomosis to the heel of the flap vessel, which is a high-risk site for blood leakage.

In this study, we evaluated the clinical outcomes of free flaps created with large-to-small venous anastomoses using the MPETS technique for soft tissue defects in the extremities.

## Patients and methods

This study was approved by our institutional ethics committee (2021–061). We examined 24 free flaps in 23 patients who received a large-to-small venous anastomosis created using the MPETS technique in soft tissue defects in the extremities between April 2015 and March 2022. Twelve patients were men and 11 were women, and their mean age was 57 (range 35–85) years. A large-to-small anastomosis was defined as a vessel size discrepancy >1.3-fold.^9^ In cases with two venous anastomoses in each flap, only flaps for which both venous anastomoses had >1.3-fold discrepancy were included in this study. The medical records were evaluated retrospectively to obtain patient demographics, flap details, details of the vessel anastomoses, and flap outcomes and complications.

### Large-to-small venous anastomosis created using the MPETS technique

**Fig. 1:**
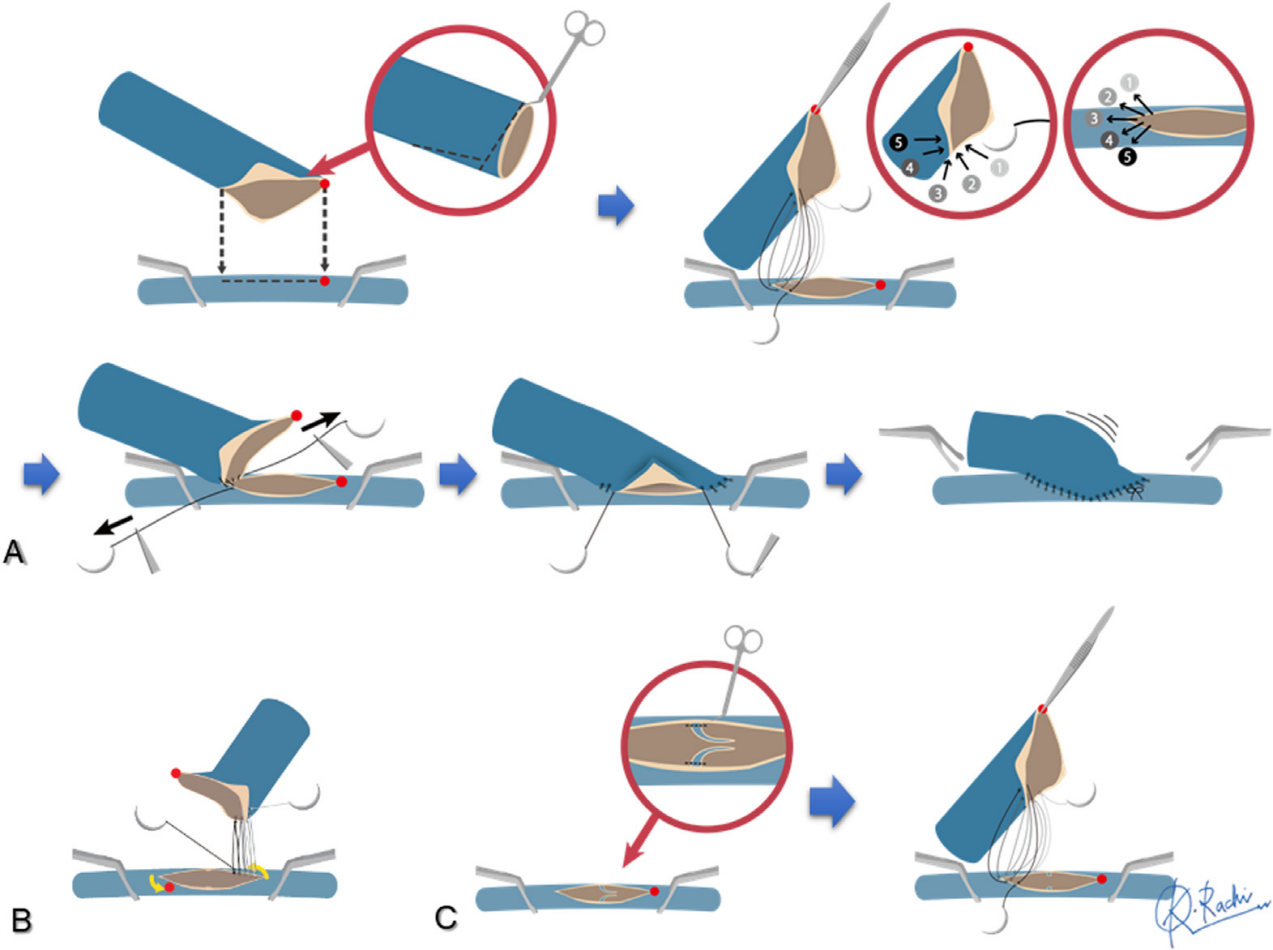
**A:** Large-to-small venous anastomosis using the MPETS technique. **B:** Shifting of the heel position of the flap vein to avoid creating a kink in the anastomotic site. **C:** Trimming of the venous valve at the anastomotic site.

**Fig. 2:**
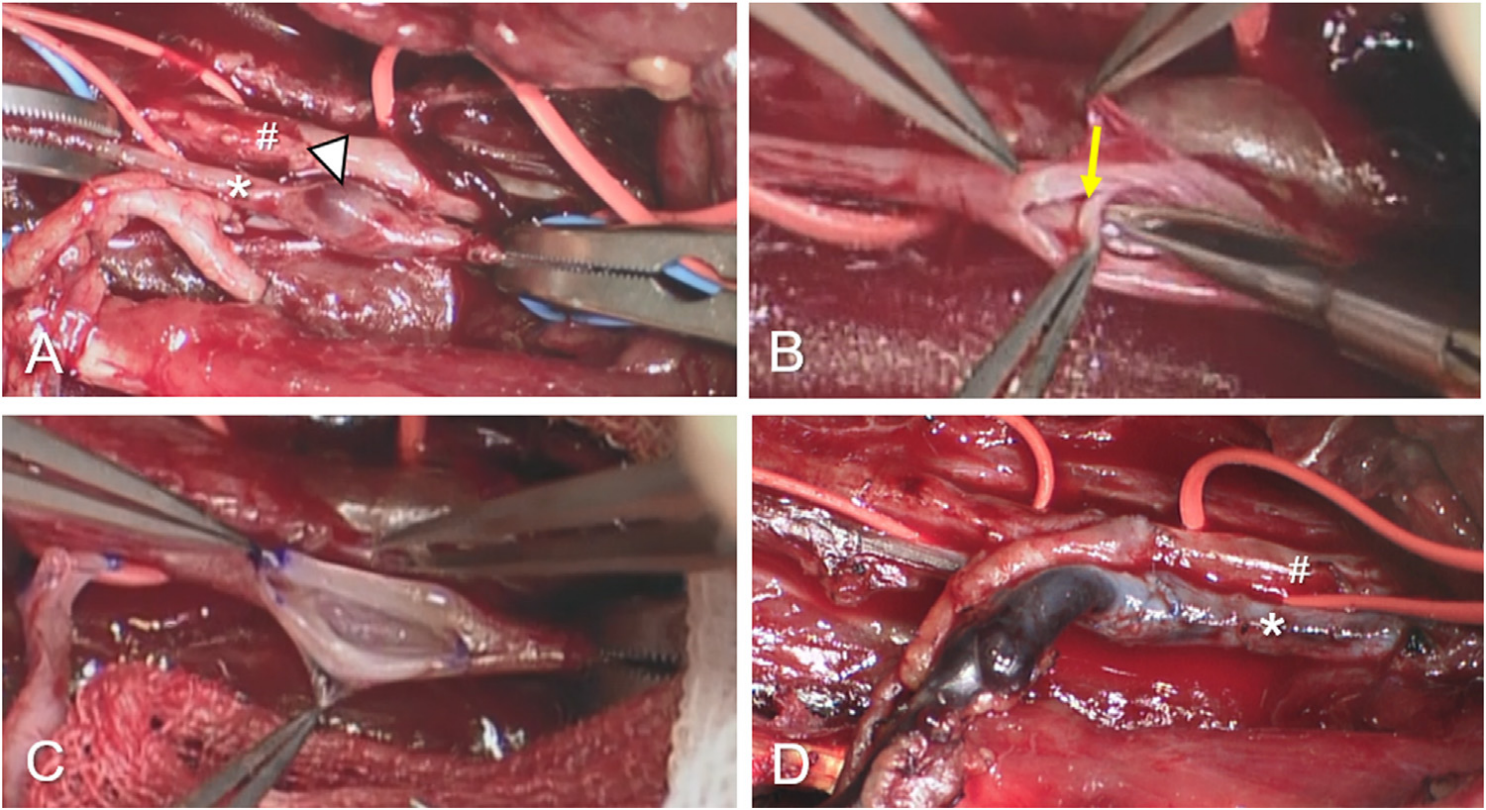
The venous anastomosis using the MPETS technique at the site of the venous valve. **A:** Tibialis posterior artery (hashmark) and accompanying vein (asterisk). The most suitable site for anastomosis was on a venous valve (arrowhead). **B:** Venous valve cusps (arrow) made it difficult to identify the vessel lumen. **C:** The MPETS technique could be performed after careful resection of the valve cusps. **D:** Finding the anastomotic site after completion of the large-to-small venous anastomosis using the MPETS technique.

The large-to-small venous anastomosis was performed using the MPETS anastomosis technique essentially as reported previously^15^^,^^16^ (Fig. 1A). In brief, the stump of the flap vein was cut obliquely into a diamond shape. The anastomosis was performed using a special, small, double-needle microsuture comprising 10 cm 9–0 suture and a 7 cm 10–0 suture, made of polyvinylidene fluoride (Asflex; Crownjun Kono Co. Ltd., Tokyo, Japan). The venotomy involved a slit shape created using a microknife and microscissors about the same size as the major axis of the widened flap vein. At first, the flap vein was tethered at the heel using a parachute technique, and this was followed by gently pulling up the ends of the sutures to draw the flap vein into the heel of the recipient vein. The posterior and anterior walls were next sutured in a continuous fashion. If the flap vein length was short and the anastomotic angle between the flap vein and recipient vein was large horizontally, the heel of the flap vessel was shifted horizontally to avoid the creation of a kink in the anastomotic site, as shown in Fig. 1B. If a venous valve was present at the suitable anastomotic site, the cusps were trimmed through a wide-slit window created on the valve while taking care not to damage the intima. After resection of the venous valve, the MPETS anastomosis was performed at the site of the venous valve (Figs. 1C and 2) (See Supplemental Online Video).

**Fig. 3:**
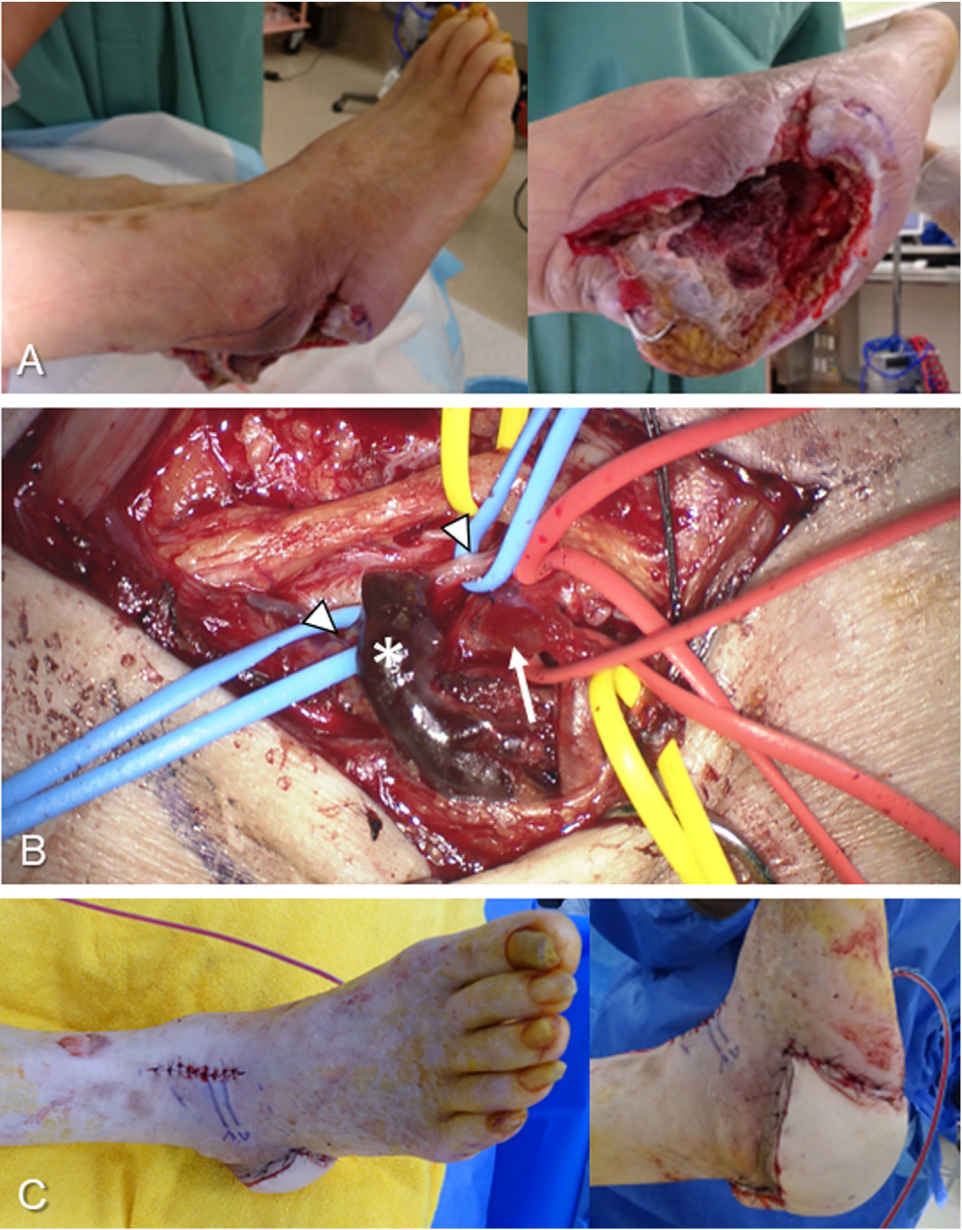
A representative case of a large-to-small venous anastomosis: a 72-year-old man presented with diabetes mellitus gangrene accompanied by osteomyelitis of the calcaneus in the right foot. **A:** After three debridements, the calcaneus was exposed through the extensive soft tissue defects. A latissimus dorsi myocutaneous flap was harvested, and flap pedicle vessels were passed into the dorsal window of the ankle joint through the lateral subcutaneous tunnel. **B:** The 4.0 mm large flap vein (asterisk) was anastomosed to the 1.3 mm small recipient vein (arrowhead) accompanying the dorsalis pedis artery with a 5.5 mm venotomy using the MPETS technique. The arrow represents the thoracodorsal artery. **C:** The flap survived without venous congestion, and the patient could walk without a cane.

For venous anastomoses for the repair of soft tissue defects caused by severe trauma or infection, reliable accompanying veins were chosen as recipient veins regardless of the vessel diameter (Fig. 3). Superficial veins with good flow were sometimes selected as recipients for the repair of soft tissue defects in fingers and hands. For arterial anastomoses, a flap artery was anastomosed with each recipient artery using either the ETE or ETS technique.

As a postoperative protocol, prostaglandin E1 was administered continuously at 80 μg/day until postoperative day 4 and then at 40 μg/day until day 7. Bed rest was ordered for 1 week, and smoking was prohibited for at least 3 weeks after the operation.

### Analysis of vessel size

The size of the venotomy was measured just after the incision was made. When the flap pedicle vessel was expanded to permit sufficient blood flow after the completion of the anastomosis, the external diameters of the flap and recipient veins were measured manually by the surgeon and assistant using an intraoperative ruler. The flap vein diameter (a), recipient vein diameter (b), length of the venotomy (c), vessel size discrepancy between the flap vein and recipient vein diameters (a/b), and expansion rate of the flap vein (c/a) were analysed for each venous anastomosis. Pearson's correlation coefficient was used to identify significant correlations between the flap vein diameter and expansion rate of the flap vein. A *p* value <0.05 was considered to be significant. This analysis was performed using BellCurve for Excel (version 3.21, Social Survey Research Information Co., Ltd., Osaka, Japan).

## Results

### Flap and anastomosis details

The patient demographics and flap details are summarized in Table 1. Of the 23 patients, 15 had at least one comorbidity; 10 had diabetes mellitus, and six had hypertension. Nine flaps were used to repair soft tissue defects associated with infection, and 14 were indicated because of trauma. The locations of the soft tissue defects were 10 in the upper extremities and 14 in the lower extremities. An anterolateral thigh flap was used in 14 flaps, and the latissimus dorsi musculocutaneous flap was used in 6 flaps.

**Table 1:** Patient demographics and flap details.

Number of patients	23		
Total numbers of flaps	24		
Sex	12	Men	
	11	Women	
Mean age, years, ± standard deviation (range)	57 ± 14	(35–85)	
Number of patients with comorbidities	15		
		10	Diabetes mellitus
		6	Hypertension
		1	Haemodialysis
		2	Hyperlipidaemia
Oral anticoagulant	2		
Current smoking	8		
Aetiology of the defect	13	Trauma	
	9	Infection	
	1	Post-traumatic scar	
	1	Flap donor	
Defect location	10	Upper extremity	
		1	Elbow
		3	Forearm
		3	Hand
		3	Finger
	14	Lower extremity	
		1	Knee
		2	Lower leg
		10	Foot
		1	Toe
Flap type	6	LD	
	14	ALT	
	1	Toe-to-finger	
	1	DUAP	
	1	Peroneal	
	1	Scapula	

LD = latissimus dorsi myocutaneous flap, ALT = anterolateral thigh flap, DUAP = distal ulnar artery perforator flap.

### Anastomosis details

**Fig. 4:**
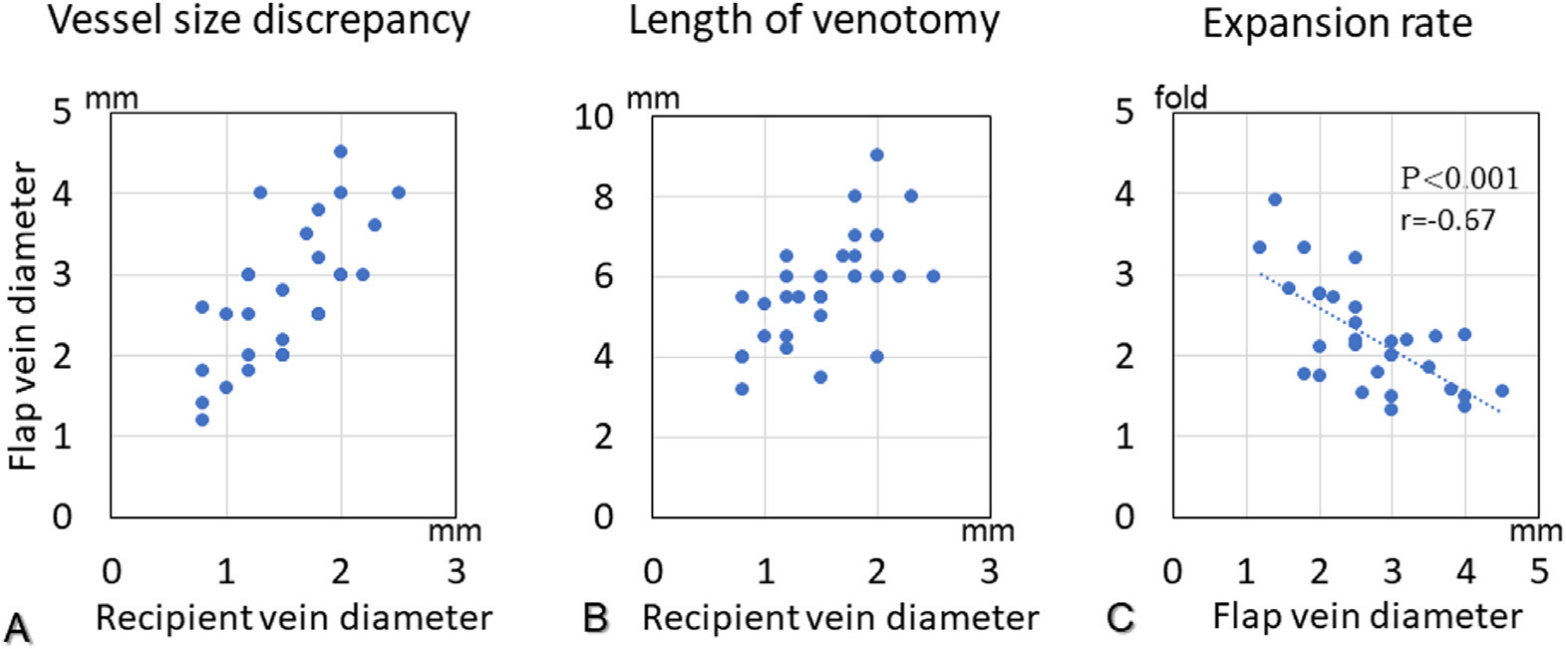
**A:** Vessel size discrepancy. **B:** Length of the venotomy. **C:** Expansion rate of the flap vein.

A total of 30 anastomosed veins were included in this series. One vein was anastomosed in 18 flaps, and two veins were anastomosed in six flaps (Table 2). We chose 24 deep veins, all of which accompanied main arteries, as recipient veins, and 6 superficial veins for the upper extremities. The mean diameters were 1.5 mm (range 0.8–2.5 mm) in the recipient veins and 2.7 mm (range 1.2–4.5 mm) in the flap veins. The mean vessel size discrepancy between the recipient and flap veins was 1.8-fold (range 1.3–3.3 fold) (Fig. 4A). The mean length of the venotomy was 5.7 mm (range 3.2–9.0 mm) (Fig. 4B), and the mean expansion rate of the flap vein was 2.2-fold (range 1.3–3.9 fold). The flap vein diameter and the expansion rate of the flap vein correlated significantly inversely (Fig. 4C).

**Table 2:** Details of the recipient arteries and anastomoses.

Number of arterial anastomoses	24		
Recipient artery	10	Upper extremity	
		2	Brachial
		5	Radial
		1	Palmar arterial arch
		2	Digital
	14	Lower extremity	
		1	Femoral
		1	Tibialis anterior
		2	Tibialis posterior
		10	Dorsalis pedis
The number of venous outflow vessels in each flap	18	1 Vein	
	6	2 Veins	
Total number of venous anastomoses	30		
Recipient venous selection	24	Deep vein (artery accompanying vein)	
	6	Superficial vein	
Recipient vein diameter (mm)	1.5 ± 0.5 (0.8–2.5)		
Flap vein diameter (mm)	2.7 ± 0.8 (1.2–4.5)		
Vessel size discrepancy (fold)	1.8 ± 0.5 (1.3–3.3)		
Length of venotomy (mm)	5.7 ± 1.4 (3.2–9.0)		
Expansion rate of flap vein (fold)	2.2 ± 0.7 (1.3–3.9)		
Shifting of the heel position of the flap vein to avoid creating a kink in the anastomotic site	3		
Trimming of a venous valve at the anastomotic site	6		
Additional stitches to control blood leakage at the anastomotic site	4		

Data are presented as mean ± standard deviation (range).

Three veins required shifting of the heel position of the flap vein to avoid kinking. Trimming of venous cusps was performed in six veins because of the presence of venous valves at the anastomotic site. Four anastomoses needed additional stitches to control blood leakage at the anastomotic site, but all leakage was easily controlled by tightening the suture around the bleeding site and tying the loosened suture with additional stitches.

### Flap outcomes and complications

One arterial and one venous thrombosis occurred in this series, but all flaps survived (Table 3). Venous congestion occurred in one case involving a latissimus dorsi myocutaneous flap for treating an extensive soft tissue defect caused by an open forearm fracture. In this case, the vein accompanying the brachial artery proximal to the antecubital fossa was chosen as the recipient vein to avoid the zone of injury, and the vessel size discrepancy was 1.4-fold. Although a complete venous anastomosis was performed, to avoid stretching the anastomotic vessel because of a short venous pedicle length, the elbow joint was fixed in a flexion position. However, 4 days after the flap surgery, venous congestion, probably caused by accidental elbow extension, was noted, and a venous reanastomosis with a venous graft was needed. One anterolateral thigh flap in another patient had arterial congestion requiring arterial reanastomosis. Both of these flaps recovered completely after vascular reanastomoses.

**Table 3:** Flap outcomes and complications.

Number of flaps that survived	24	
Flap-related complications	1	Venous congestion requiring venous reanastomosis with vein graft
	1	Arterial congestion requiring arterial reanastomosis
	1	Partial flap loss: 1 LD flap that was not fully covered by the angiosome
	7	Deep wound infections
Additional surgery except for vascular problems	8	Debridements under the flap
	5	Defatting
	4	Secondary closures
	3	Implant removals
	2	Bone grafts
	2	Tendon transfer
	1	Split-thickness skin graft
	1	Web-plasty

LD = latissimus dorsi myocutaneous flap.

One patient with a latissimus dorsi myocutaneous flap developed partial flap necrosis possibly because the angiosome did not fully cover the flap and defect. Postoperative deep infection occurred in seven patients: in three of these patients because of extensive crushed injury, in three patients because of diabetes mellites gangrene, and in one patient because of postoperative osteomyelitis. Debridement under the flap was needed for eight patients, including one with suspected infection. The soft tissue defects in these eight patients healed after thorough debridement, and the function of the extremities was preserved.

## Discussion

Because of a limited supply of reliable and suitably sized veins for recipient vessels in free flap surgery for soft tissue defects caused by severe trauma and infection, it is important to manage vessel size discrepancy between the recipient and flap veins.^2^^,^^17^^,^^18^ The surgeon sometimes has no choice but to select a small vein as a recipient vein even though this can make a venous anastomosis difficult and complicated.^9^ The previously reported MPETS technique is simple, reliable, and useful for various types of anastomoses in free flap surgery, regardless of a vessel size discrepancy. However, there are few reports of the ETS procedure performed using a wide-slit venotomy and/or parachute technique for a complicated venous anastomosis. In this study, we found few complications related to the venous anastomosis involving >1.3-fold vessel size discrepancy between the recipient and flap veins, and all flaps survived. The MPETS technique may be a good option for the venous anastomosis of a large flap vein to a small recipient vein.

The conventional ETS technique for venous anastomosis is useful for large recipient veins such as the internal cervical vein.^19^, ^20^, ^21^ Some advantages of venous ETS compared with ETE have been reported, including the ease of adjusting for the vessel size discrepancy, the ease of judging the recipient vessel reliability from backflow through the venotomy window, and the preservation of the peripheral circulation to prevent venostasis and increase the force driving venous return.^9^^,^^12^^,^^22^ However, the ETS technique for venous anastomosis has been reported to be more difficult than ETE, and the usefulness of the ETS technique for venous anastomoses remains unclear.^23^^,^^24^

The previously reported MPETS technique has the following three distinct advantages over the conventional ETS technique^15^^,^^16^: (1) the ease of performing a vesselotomy by making only a wide-slit window; (2) the avoidance of anastomotic narrowing by making a very wide-slit vesselotomy into the recipient vessels and by attaching a diamond-shaped stump of a flap pedicle into its window to keep it open, as in a vascular stent; and (3) the ease of performing the anastomosis at the heel of the donor vessel, which is a high-risk site for blood leakage. The MPETS technique may also be applied to very small recipient veins under microscopy when reliable suturing can be performed while looking into the vessel lumen through the wide-slit window. The one case of venous congestion in this series caused by pedicle kinking because of a short pedicle length indicates that it is essential to harvest a sufficient length of the flap vein when performing the MPETS procedure.

When suitably sized veins for recipient veins cannot be found around the soft tissue defect, the surgeon could seek more reliable and larger veins by making additional incisions in proximal parts of the defect and anastomosing the flap vein into its proximal recipient vein with or without a vein graft. However, for the surgeon skilled in performing large-to-small venous anastomosis, small veins accompanying main arteries can be used as recipient veins without having to make an intraoperative decision about selecting the recipient veins and without additional incisions to seek proximal veins.^7^ Accompanying veins, especially in the lower extremities, are often small, and large-to-small venous anastomosis seems to be more important,^8^^,^^9^ as observed in 11 of our patients who had veins accompanying the tibialis anterior artery or dorsalis pedis artery were used as recipient veins.

The optimal size of the venotomy remains unclear in the MPETS technique for large-to-small venous anastomosis. In this study, the mean venotomy length was 5.7 mm, which was much larger than the mean diameter of 1.5 mm for recipient veins. When using the MPETS technique, we believe that it is important to create a wide window to be able to see clearly into the lumen of the recipient vessel during each stitch. In our study, the expansion rate of the flap vein correlated inversely and significantly with the diameter of the flap vein (Fig. 4C). When a large flap vein is anastomosed into a smaller recipient vein using the ETS technique, a large expansion of the flap vein may not be necessary if the lumen of the recipient vessel can be seen clearly through the venotomy window.

For a venous anastomosis during free flap surgery, the presence of a venous valve in the recipient vessel is considered to contribute to venous congestion, and the surgeon may need to change the decision about the anastomotic site.^25^^,^^26^ In free flap surgery for head and neck reconstruction, the outcome is excellent using the ETS technique with the internal jugular vein as the recipient vein, but the presence of venous valves has not been mentioned in previous reports.^19^, ^20^, ^21^ Venous valves are found at a higher frequency, and each venous cusp is longer in the extremities than in the trunk.^27^ Because the venous cusp can be trimmed carefully through a large window without damaging the intima (Figs. 1C and 2), the venous anastomosis can be performed on the venous valve with the MPETS technique. Venous valve resection is commonly performed during in situ distal bypass surgery and results in good long-term patency.^28^^,^^29^ We found no complications, such as venous thrombosis or peripheral oedema related to venous valve resection in the six patients in our study. Given the possibility of finding a venous valve at the anastomotic site when using the ETS technique with a wide-slit venotomy, including the MPETS, the surgeon should be prepared to deal with a venous valve.

Our study has some limitations. First, this was a retrospective study and included a small sample. Second, there was no control group. Therefore, we cannot conclude whether the MPETS was superior to the conventional ETE and ETS techniques for venous anastomosis.

## Conclusions

We evaluated the clinical outcomes of free flaps created with large-to-small venous anastomosis using the MPETS technique in soft tissue defects of the extremities. Because of the limited availability of reliable and suitably sized veins for free flap surgery for the repair of soft tissue defects caused by severe trauma or infection, it is important to deal properly with vessel size discrepancy between the recipient and flap veins. The MPETS technique is simple, reliable, and useful for performing various types of venous anastomoses, regardless of vessel size discrepancy and the presence of a venous valve. This technique may be a good option for venous anastomosis with vessel size discrepancy between a small recipient vein and a large flap vein.

## Conflicts of interest statement

None.

## Appendix Supplementary materials

Image, video 1
